# Social support plays a role in the attitude that people have towards taking an active role in medical decision-making

**DOI:** 10.1186/s12913-016-1767-x

**Published:** 2016-09-21

**Authors:** Anne E. M. Brabers, Judith D. de Jong, Peter P. Groenewegen, Liset van Dijk

**Affiliations:** 1 NIVEL, the Netherlands Institute for Health Services Research, PO Box 1568, 3500 BN Utrecht, The Netherlands; 2 Department of Sociology, Utrecht University, PO Box 80125, 3508 TC Utrecht, The Netherlands; 3 Department of Human Geography, Utrecht University, PO Box 80125, 3508 TC Utrecht, The Netherlands

**Keywords:** Medical decision-making, Patient participation, Social networks, Social support

## Abstract

**Background:**

There is a growing emphasis towards including patients in medical decision-making. However, not all patients are actively involved in such decisions. Research has so far focused mainly on the influence of patient characteristics on preferences for active involvement. However, it can be argued that a patient’s social context has to be taken into account as well, because social norms and resources affect behaviour. This study aims to examine the role of social resources, in the form of the availability of informational and emotional support, on the attitude towards taking an active role in medical decision-making.

**Methods:**

A questionnaire was sent to members of the Dutch Health Care Consumer Panel (response 70 %; *n* = 1300) in June 2013. A regression model was then used to estimate the relation between medical and lay informational support and emotional support and the attitude towards taking an active role in medical decision-making.

**Results:**

Availability of emotional support is positively related to the attitude towards taking an active role in medical decision-making only in people with a low level of education, not in persons with a middle and high level of education. The latter have a more positive attitude towards taking an active role in medical decision-making, irrespective of the level of emotional support available. People with better access to medical informational support have a more positive attitude towards taking an active role in medical decision-making; but no significant association was found for lay informational support.

**Conclusions:**

This study shows that social resources are associated with the attitude towards taking an active role in medical decision-making. Strategies aimed at increasing patient involvement have to address this.

## Background

Patients traditionally delegate decision-making to physicians because they trust that physicians take decisions based both on scientific evidence and on what is best for an individual patient ([1], p7-8). As physicians control most of the medical decisions, professional judgements rather than collaborative decisions including patients’ own preferences often determine which treatment a patient receives ([1], p9). However, this paternalistic model, has come to be questioned in the past decades. At the same time, the position of patients in health care has altered significantly, at least in theory. Patients are supposed to take an active role in their health [2], and they are expected to be involved in decisions about their health [3]. The right of patients to engage in these decisions has been enshrined in the laws of several countries [4]. In the Netherlands, the setting for this study, the approach of patient participation in decision-making is formally defined in the Medical Treatment Agreement Act (WGBO) [5]. There is, thus, a growing emphasis on involving patients in medical decision-making. Providing care that is respectful of, and responsive to, an individual patient’s preferences, is regarded as one of the aspects of good quality of care [6].

Most patients prefer to be involved in medical decision-making [7]. Moreover, it has been found that preferences for involvement, as well as actual involvement in decision-making, have increased over time [7, 8]. On the other hand, it has been recognized that categories of patients prefer to leave the decision to their physician [9]. Among others, diagnosis, health status and characteristics of the patient affect whether patients prefer to be involved in decision-making [10]. For example, in a situation where patients are acutely ill or incapacited, they generally have to delegate the decision-making process to their physician [11, 12]. Patient characteristics are associated with preferences regarding decision-making. Several studies consistently found that younger people, higher educated people, and women want, more often, an active role in decision-making [9, 10, 13].

However it is not only patient characteristics which have to be taken into account in explaining whether patients want to participate in medical decision-making, but also has a patient’s social context. The reason for this is that patients’ preferences cannot be interpreted as merely individual. Social resources, as well as social norms affect individual behaviour [14–16]. Therefore, in this study, we aim to examine the role of social resources in relation to whether patients want to have an active role in medical decision-making. To our knowledge, this has not yet been studied. By investigating patients in the Netherlands, we aim to answer the following research question in this study: How are a patient’s social resources associated with taking an active role in medical decision-making? We focus on the *attitude* towards taking such an active role role in medical decision-making.

## Theory and hypotheses

Within an individual’s social contexts, such as their work, family, or neighbourhood, individuals meet members of their social network. Someone’s social network refers to the web of social relationships surrounding this person [14]. Social relationships influence health – and health behaviour – by different mechanisms [15, 17]. They can create social norms, as well as provide resources that affect behaviour [14–16]. In this study, we focus specifically on how resources provided by someone’s social network affect the attitude towards taking an active role in medical decision-making.

The pool of resources residing in members of an individual’s social network form an individual’s social capital, or social resources [18, 19]. Social resources can be provided in the form of social support [20]. The literature provides multiple interpretations of the concept of social support [21]. We will use the framework of Tardy [21], who argues that social support consists of five aspects:

1. Network - as mentioned before, the social network of a patient serves as a source of support.
2. Direction - social support can be both given and received. We opt to examine social support from the direction of the *recipient*.
3. Disposition - social support can be both available and received. We focus here on an attitude towards behaviour, and therefore on the *availability* of support. “Support availability refers to the quality or quantity of support to which people have access” ([21], p188).
4. Description or evaluation - since we focus on the availability of support, we examined the description of social support, that is the degree to which social support is available.
5. Content - Often distinguished types of social support are: emotional, instrumental, and informational support. Emotional support includes providing empathy, listening, and giving advice. Instrumental support refers to the tangible help that others may provide, for example, offering money, transport and time. Informational support is the help others may give through the provision of information [20, 22, 23]. We think that for participation in medical decision-making, the availability of both informational and emotional support are important. Informational support can be related to the provision of advice about different treatments. With respect to emotional support, we expect that support, especially in the form of being accompanied during the medical consultation, is relevant. People who accompany others to such consultations – called companions – play an important role in providing emotional support [24]. We argue that instrumental support is less relevant in our context. One reason for this is that in the Netherlands the costs for most medical care, medicines and medical devices are covered by the basic health insurance package [25], and also because there is a low level of out-of-pocket payments [26]. Furthermore, the aspects of time and transport, for example having someone who bring you to the consultation, is already included, since we focus on emotional support in the form of being accompanied during the consultation.

To summarize, we focus on how the availability of emotional and informational support is associated with the attitude towards taking an active role in medical decision-making.

### Hypotheses

Traditionally patients have left the decision-making process to their physician. One reason for this might be that patients who are seriously ill feel vulnerable, and therefore cannot, or do not want to, take the responsibility of being involved in medical decision-making [27, 28]. Another reason might be the information asymmetry between physicians and patients: physicians have information that patients do not [29]. It has been acknowledged that patients believe that medical decision-making requires specific knowledge that they do not have [30] and therefore they leave the decision to their physician. We expect that this lack of knowledge can be compensated by getting advice from others – that is informational support – and by receiving emotional support. By receiving informational support patients acquire specific knowledge required for participating in medical decision-making. Patients can receive this information from health care professionals in their social network – that is medical informational support – as well as from lay people in their network – that is lay informational support. Consequently, we expect that the more medical and lay informational support people have available, the more positive their attitude will be towards taking an active role in medical decision-making.

In the context of our study, emotional support can be provided by accompanying the patient during the consultation. We expect that patients will feel less vulnerable due to receiving emotional support. The reason for this is that it has been suggested that patients feel more confident when a companion is present [31]. Furthermore, comparable to informational support, emotional support also has a role in acquiring information necessary for medical decision-making. It has been suggested that having a companion present during the consultation can support the interaction between the patient and the physician by supporting the patient’s communication. For example by asking the patient questions, prompting the patient to talk, and asking for the patient’s opinion. In addition physicians are more informative when a companion is present [31–33]. Furthermore, companions remember information, which is likely to benefit the patient [34]. As a result, we expect that patients who have more emotional support available take a more positive attitude towards taking an active role in medical decision-making.

- H1: The more medical informational support people have available in their social network, the more positive their attitude is towards taking an active role in medical decision-making
- H2: The more lay informational support people have available in their social network, the more positive their attitude is towards taking an active role in medical decision-making
- H3: The more emotional support people have available in their social network, the more positive their attitude is towards taking an active role in medical decision-making

People also possess personal resources, such as knowledge and skills in, for example, communication and numeracy. We expect that the relationship between social support and the attitude towards taking an active role in medical decision-making will differ between people possessing more or less of this knowledge and skills. More specifically, we hypothesize that informational support is of less value for people with more knowledge and skills, as there is less information asymmetry between them and the physician compared to people with less knowledge and skills. For example, higher educated people receive more information from physicians than lower educated people [35]. We also expect that for people with more knowledge and skills, the role of a companion, especially in the function of aquiring more information, is of less value. The reason for this is that higher educated people are not only more assertive and expressive, but also ask more questions themselves [35]. This allows them to acquire more knowledge necessary for medical decision-making. We thus hypothesize that the role of emotional support in taking an active role in medical decision-making is less important for people with more knowledge and skills.

- H4: The role of medical informational support on the attitude towards taking an active role in medical decision-making is more important for people with less knowledge and skills compared to people with more knowledge and skills.
- H5: The role of lay informational support on the attitude towards taking an active role in medical decision-making is more important for people with less knowledge and skills compared to people with more knowledge and skills.
- H6: The role of emotional support on the attitude towards taking an active role in medical decision-making is more important for people with less knowledge and skills compared to people with more knowledge and skills.

**Fig. 1:**
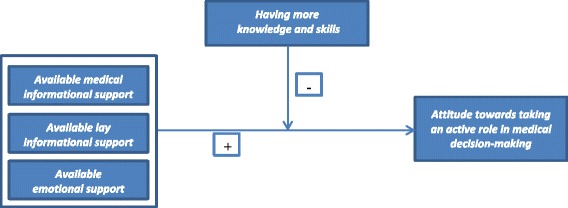
Theoretical model tested within this study

Based on the theory and hypotheses proposed, Fig. 1 presents the model tested in this study.

## Methods

### Setting

Data were collected through the Dutch Health Care Consumer Panel [36]. This panel aims to measure the attitude towards, and knowledge of, health care, as well as the expectations and experiences of health care among a cross-section of the Dutch population. The Consumer Panel is a so-called access panel. An access panel consists of a large number of people who have agreed to answer questions on a regular basis. In addition, many background characteristics, for example age, gender, and the level of education of these people are known. At the time of the study (June 2013), the access panel consisted of approximately 8500 people aged 18 years and older. Each individual panel member receives a questionnaire approximately three times a year and can quit the panel at any time. There is no possibility for people to sign up for the panel on their own initiative. The Consumer Panel is renewed on a regular basis. Renewal is necessary to make sure that members do not develop specific knowledge of, and attention for, health care issues, and that no ‘questionnaire fatigue’ occurs. Moreover, a system of renewal compensates for panel members who, for example, have died or moved without providing a forwarding address. All panel members included in this study were recruited in the spring of 2013 via seven general practices (GPs) participating in the NIVEL Primary Care Database (see this website http://www.nivel.nl/nl/NZR/zorgregistraties-eerstelijn for more information). Data are processed anonymously, and the data collection is registered with the Dutch Data Protection Authority (nr. 1262949). In addition, a privacy regulation is available for the Consumer Panel. According to the Dutch legislation, neither obtaining informed consent nor approval by a medical ethics committee, is obligatory for carrying out research in the panel (see this website http://www.ccmo.nl/en/your-research-does-it-fall-under-the-wmo).

### Questionnaire

We sent the self-administered questionnaire to a sample of 1854 panel members, recruited from these seven practices, early in June 2013. According to their preference, stated previously, 765 members received a questionnaire by post and 1089 through the internet. Panel members were free to answer the questions or not.

Two electronic reminders (after 1 and after 2 weeks), and one postal reminder (after 2 weeks) were sent to panel members who had not yet responded. The closing date for the questionnaire was 4 weeks after the initial sending. The questionnaire was returned by 1300 panel members (response rate 70 %).

### Measures

#### Dependent variable

We used two propositions of Flynn et al. [37] to measure the attitude towards taking an active role in medical decision-making. They argue that preferences towards medical decision-making may be different depending on the nature of a particular decision [37]. The authors performed their study among a population-based sample of older adults, and therefore included four broad-spectrum propositions rather than specific propositions [37]. These four propositions were based on the three phases of the framework of Charles et al. [11, 38]: information exchange, deliberation and deciding on which treatment to implement, as well as on the Autonomy Preference Index [39]. Because we also performed our study among a population-based sample, we decided to use the propositions of Flynn et al. [37]. Since our study focuses on decision-making around treatment, we only included the two propositions that concern this subject. These two propositions were: 1) *‘I would rather have my doctor make the decisions about what’s best for my health than to be given a whole lot of choices’*; 2) *‘The important medical decisions should be made by my doctor, not by me’*. The propositions were translated from English to Dutch. The propositions were scored on a 5-point Likert scale, ranging from strongly disagree (score 1) to strongly agree (score 5). We evaluated whether the two propositions measured a single concept by calculating the internal consistency given by Cronbach’s alpha. We recoded both propositions (i.e. 5 = 1, 4 = 2 etc). Moreover, we only included the respondents who filled out both propositions (included *N* = 1285; excluded *N* = 15). The internal consistency was good (alpha 0.84). Subsequently, a mean score was calculated ranging from 1 to 5, in which higher scores indicated a more positive attitude towards taking an active role in medical decision-making.

#### Independent variables

Several instruments exist to measure social support, however, these instruments did not fit within the context of our study, namely the availability of support relevant for medical decision-making. Therefore, we decided to develop our own propositions.

##### The availability of medical informational support

To assess whether people have medical informational support available in their social network, we asked them if they know people who practice one of the following medical professions: general practitioner (GP), medical specialist, and nurse. We asked respondents to indicate, for all three medical professions, whether they know someone, with the following options: ‘yes, my partner is’, ‘yes, my child is’, ‘yes, someone else is’, ‘I don’t know a’. We clarified that it was not about the respondent’s own GP, medical specialist, or nurse. For each proposition, multiple answers were possible, however, the option, ‘I don’t know a…’ could not be filled out with the other three answers. We scored, for each of the three medical professions, whether a respondent knows at least someone practicing that profession (1), or not (0). We evaluated whether these three propositions measured a single concept by calculating the internal consistency given by Cronbach’s alpha (α 0.65). Factor analysis of the data identified one factor. Thereafter, we summed up the scores of the three propositions to construct a scale for the availability of medical informational support ranging from 0 to 3, in which a higher score indicates more access to medical informational support. We only included respondents who filled out all three propositions (included *N* = 1146; excluded *N* = 154).

##### The availability of lay informational support

We presented six propositions to assess the availability of lay informational support which respondents may enjoy in their social network. The propositions were: Who would you 1) *‘involve in seeking information regarding your medical condition and/or what test results mean for you?’*; 2) *‘involve in understanding the information gathered about your condition and/or test results?’*; 3) *‘involve in seeking information about different treatment options?’*; 4) *‘involve in understanding the information gathered about the different treatment options?’*; 5) *‘involve in making a choice for one of the treatment options?’*; 6) *‘ask if he or she agrees with your choice of treatment?’*. We asked respondents to indicate, for each proposition, who they would involve in that step, with the following options: ‘partner’, ‘child’, ‘someone else’, and ‘nobody’. We instructed the respondents that it was not about common complaints, but about more severe ones. For each proposition, multiple answers were possible, however, the option ‘nobody’ could not be filled out with the other three options. For each proposition, we scored whether a respondent would involve at least someone (1), or nobody (0). We evaluated whether these six propositions measured a single concept by calculating the internal consistency given by Cronbach’s alpha. Factor analysis of the data identified one factor and the internal consistency was good (Cronbach’s α 0.88). Thereafter, we summed up the scores of the six propositions in order to construct a scale for the availability of lay informational support ranging from 0 to 6, in which a higher score indicates more access to lay informational support. We only included respondents who filled out all six propositions (included *N* = 1269; excluded *N* = 31).

##### The availability of emotional support

We presented four propositions in order to assess the emotional support respondents have available in their social network. The propositions were: Who would you 1) *‘take with you to a medical consultation where you explained your symptoms?’*; 2) *‘take with you to a medical consultation where you heard the results of medical tests?’*; 3) *‘take with you to a medical consultation where you were told about the different options for treatment?’*; 4) *‘take with you to a medical consultation where you discussed with your physician your different options for treatment?’*. We asked respondents to indicate for each proposition who they would involve in that step, with the following options: ‘partner’, ‘child’, ‘someone else’, and ‘nobody’. We instructed the respondents that it was not about common complaints, but about more severe complaints. For each proposition, multiple answers were possible, however, the option ‘nobody’ could not be filled out with the other three options. For each proposition, we scored whether a respondent would involve at least someone (1), or nobody (0). We evaluated whether these four propositions measured a single concept by calculating the internal consistency given by Cronbach’s alpha. Factor analysis of the data identified one factor and the internal consistency was good (Cronbach’s α 0.81). Thereafter, we summed up the scores of the four propositions in order to construct a scale for the availability of emotional support ranging from 0 to 4, in which a higher score indicates more access to emotional support. We only included respondents who filled out all four propositions (included *N* = 1276; excluded *N* = 24).

#### Interaction variable

Knowledge and skills was operationalized by the highest level of education completed. The level of education was classified as low (none, primary school or pre-vocational education) (0), middle (secondary or vocational education) (1), and high (2) (professional higher education or university).

#### Control variables

We included age (continuous), gender (0 = men, 1 = women), and whether the respondent filled out the questionnaire through the internet (1), or by post (0) as control variables.

### Statistical analyses

Firstly, we performed descriptive analyses. By one-way analyses of variance, we tested whether the attitude towards taking an active role in medical decision-making differed between the seven GPs from which the respondents included in this study were recruited. If there were differences between the seven GPs then we had to take these into account throughout the rest of our analyses. Secondly, in order to test the hypotheses, a regression model was constructed. We constructed a lineair regression model including all the main and the interaction variables.

The interaction effects were examined to test the hypotheses that the relationship between medical decision-making and the three aspects: the emotional support available; lay informational support and medical informational support available, is modified by another mechanism (H4, H5 and H6). We subsequently removed stepwise the interaction effects that were not significant, starting with the one that was least significant, from the regression model in order to facilitate the interpretation of the other effects. In the regression analyses, categorical variables, for example, the level of education, were recoded into dummy variables. The continuous variable, age, was centred on the mean age. This ensures that 0 has a meaningful value, and that the interpretation of effects will occur at a meaningful value. The level of statistical significance was fixed at 0.05. All statistical analyses were carried out using STATA, version 13.1.

## Results

The mean age of the respondents was 56 years, ranging from 18 to 84 years, and more than half (55 %) of the respondents were women (Table 1). Almost half (47 %) had a middle level of education. Compared to the Dutch population aged 18 years and older, older people (≥65 years) were overrepresented in the group of respondents [36].

**Table 1:** Descriptive statistics of the respondents

	N		% or mean (SD)
Gender	1300		
Male		583	44.9
Female		717	55.2
Age (SD)	1300		56 (15.8)
Education	1270		
Low (none, primary school or pre-vocational education)		273	21.5
Middle (secondary or vocational education)		596	46.9
High (professional higher education or university)		401	31.6
Questionnaire	1300		
Post		571	43.9
Internet		729	56.1
Attitude towards taking an active role in medical decision-making (SD) (range 1-5, higher scores indicates a more positive attitude)	1285		3.22 (1.03)
Available medical informational support (SD) (range 0-3)	1146		1.01 (1.03)
Having no medical informational support available			39 %
Available lay informational support (SD) (range 0-6)	1269		5.40 (1.43)
Having no lay informational support available			4 %
Available emotional support (SD) (range 0–4)	1276		3.20 (1.23)
Having no emotional support available			8 %

The mean score for attitude towards taking an active role in medical decision-making was 3.22 (SD 1.03) on a scale from 1 to 5, where a higher score indicates that respondents are more positive towards taking an active role in such decisions (Table 1). One-way analyses of variance showed that the attitude towards taking an active role in medical decision-making did not differ between the seven GPs (range mean score: 3.11–3.39, *p* = 0.329). Consequently, we did not have to take into account from which GP the respondents were recruited.

The mean score for the *availability of medical informational support* was 1.0 on a scale from 0 to 3, where 3 is the highest level of medical informational support available (Table 1). Thirty-nine per cent of the respondents indicated that they do not know anyone from a medical profession. Table 1 shows that the mean score for the *availability of lay informational support* was 5.4 on a scale from 0 to 6, where 6 is the highest level of lay informational support available. Four per cent (*N* = 49) of the respondents indicated that they had no lay informational support available at all. The mean score for the *availability of emotional support* was 3.2 on a scale from 0 to 4, where 4 is the highest level of emotional support available. Eight per cent (*N* = 101) of the respondents indicated that they had no emotional support, in the form of someone to accompany them to the consultation, available at all.

### Test of the hypotheses

Table 2 shows the results of the regression analysis to test the hypotheses about the role of the availability of informational and emotional support on the attitude towards taking an active role in medical decision-making. The interaction effect between knowledge and skills - measured as the highest level of education completed - and *emotional* support was significant and therefore kept in the model. The other two hypothesized interaction effects (H4 and H5) were not significant and thus removed from the model in order to facilitate the interpretation of the other effects. The explained variance of the model was 18 % (adjusted R-square 0.175).

**Table 2:** Regression model to examine the association between the availability of informational and emotional support and attitude towards taking an active role in medical decision-making (*N* = 1089)

Attitude towards taking an active role in medical decision-making (range 1-5, higher scores indicates a more positive attitude)	Coef.	Beta^a^	*P*-value
Available medical informational support (0 = no; 3 = most)	0.106	0.106	**0.000**
Available lay informational support (0 = no; 6 = most)	−0.023	−0.032	0.352
Available emotional support (0 = no; 4 = most)	0.167	0.202	**0.002**
Available emotional support * level of education			
Low (none, primary school or prevocational education)	Reference	Reference	Reference
Middle (secondary or vocational education)	−0.184	−0.323	**0.002**
High (professional higher education or university)	−0.159	−0.251	**0.016**
Level of education			
Low (none, primary school or prevocational education)	Reference	Reference	Reference
Middle (secondary or vocational education)	1.100	0.534	**0.000**
High (professional higher education or university)	1.381	0.632	**0.000**
Gender (0 = man; 1 = woman)	0.111	0.054	0.057
Age (centred around mean age)	−0.008	−0.117	**0.000**
Questionnaire (1 = internet; 0 = post)	0.167	0.081	**0.005**
Constant	2.019	-	**0.000**

Adjusted R-square: 0.175

Bold type indicates *p* < 0.05

^a^Standardized coefficients

#### The association between the availability of medical and lay informational support and the attitude towards taking an active role in medical decision-making (H1, H2, H4 & H5)

In line with H1, Table 2 demonstrates that the availability of *medical informational* support is significantly associated with the attitude towards taking an active role in medical decision-making. This implies that people with more medical informational support available have a more positive attitude towards taking an active role in medical decision-making. No significant effect was observed for the availability of *lay informational* support, rejecting H2. Contrary to our hypotheses (H4 and H5), we did not find an interaction effect between both the availability of *medical and lay informational* support and educational level, as an indication for one’s own knowledge and skills.

#### The association between the availability of emotional support and the attitude towards taking an active role in medical decision-making (H3 & H6)

**Fig. 2:**
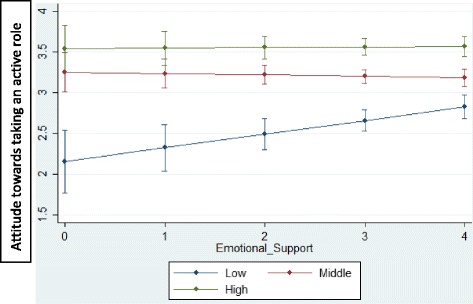
Relation between the availability of emotional support and attitude towards taking an active role in medical decision-making for people with a low, middle and high level of education

We found an interaction effect between educational level and available *emotional* support. This interaction effect means that the relationship between the availability of emotional support and the attitude towards taking an active role in medical decision-making varies by educational level, confirming H6. Further examination of this interaction effect shows that it depends on the level of education whether there is a positive relationship between the available emotional support and the attitude towards taking an active role in medical decision-making (Fig. 2). Only in cases of people with a low level of education is the available *emotional* support positively associated with the attitude towards taking an active role in medical decision-making. There is no significant relation between the available *emotional* support and the attitude towards taking an active role in medical decision-making for people with a middle and high level of education. H3 is only confirmed for people with a low educational level. The interaction effect of the available *emotional* support and the level of education also indicates that the effect of educational level on the attitude towards taking an active role in medical decision-making varies with the level of the availability of *emotional* support. Further examination of this effect demonstrates that whether educational level influences the attitude towards taking an active role in medical decision-making does not depend on the level of the availability of *emotional* support. No matter what the level of the availability of emotional support, people with a middle and high educational level have a more positive attitude towards taking an active role in medical decision-making than people with a low educational level.

### Control variables

Age is significantly associated with the attitude towards taking an active role in medical decision-making, while no significant effect is observed for gender. Respondents that filled out the questionnaire through the internet have a more positive attitude than respondents that filled out the questionnaire by post (see Table 2).

## Discussion

This study examined the association between the availability of informational (both medical and lay) and emotional support and people’s attitude towards taking an active role in medical decision-making. In line with our hypothesis, we found that people who enjoyed more available medical informational support are more positive towards taking an active role in medical decision-making. This study also showed that only for people with a low level of education the availability of emotional support was positively related to the attitude towards taking an active role in medical decision-making, partially confirming our hypothesis. We also found that no matter what level of emotional support was available, people with a middle and high level of education have a more positive attitude towards taking an active role in medical decision-making. Contrary to our hypothesis, no effect was observed for the availability of lay informational support. In line with earlier research, we found that older people have a less positive attitude towards taking an active role in medical decision-making. Finally, we found that people who filled out the questionnaire through the internet have a more positive attitude towards taking an active role in medical decision-making.

We showed that social resources play a role in the attitude towards taking an active role in medical decision-making. People with more health care professionals in their network have a more positive attitude towards taking an active role. The reason for this might be that these people are more easily able to contact a health care professional within their network that they trust in order to seek information about their medical problems and related treatment options. These professionals in their network can, for instance, inform patients about different treatment options, but also advise them on which treatment option to choose. As a result, people are better equipped with information necessary for medical decision-making. We noticed that for people with a low educational level, the availability of emotional support contributes positively to their attitude towards taking an active role in medical decision-making. This is in line with what we expected. The reason for this might be that a companion compensates for less knowledge and skills by, for example, prompting questions, as well as that people feel more confident when someone is present during the consultation.

Besides the influence of social resources, we also found a significant association between educational level and the attitude towards taking an active role in medical decision-making. Compared to people with a low level of education, middle and high educated people have a more positive attitude towards taking an active role in medical decision-making. These results confirm empirical research [9, 10, 13]. Finally, in line with earlier research [9, 10, 13], we found that older people have a less positive attitude towards taking an active role in medical decision-making.

This study gives a first insight into the role of social resources on the attitude towards taking an active role in medical decision-making. Our study confirms that someone’s social resources are related to their attitude towards taking an active role in medical decision-making. However, our model does not explain most of the variance in the attitude towards taking an active role in such decisions. This implies that other factors besides the availability of support and patient characteristics influence the attitude towards taking an active role. Social relationships influence individual behaviour through different mechanisms, of which providing resources is only one. Someone’s social network also influences individual behaviour through the creation and sharing of norms [16]. Social norms specify what actions are regarded by a group of people as normal, and what actions are regarded as deviant ([16], p242). If it is common in a social environment to leave the decision to physicians, because there is great respect towards physicians, then individuals are expected to be less likely to take an active role in the process, since this is the norm. For further research, we recommend examining the relationship between social norms and taking an active role in these medical decisions.

The highest level of education completed can be used as operationalisation for knowledge and skills. However, educational level might not completely cover this concept [40]. Another operationalisation might be someone’s health literacy skills. Health literacy is defined by the Institute of Medicine as “the degree to which individuals have the capacity to obtain, process, and understand basic health information and services needed to make appropriate health decisions” [41]. Yet, several studies have examined the relation between health literacy and involvement in medical decision-making [42–48]. Future research is recommended to further examine the relation between health literacy and involvement in medical decision-making.

Currently, decision aids (DAs) are increasingly being used to enhance patient participation in the decision-making process. A DA aims to provide patients with information about options in sufficient detail for patients to arrive at informed judgements about the personal value of those options ([49], p717). As such, patients are expected to be better equipped with the medical information required for taking a decision. A literature review shows that DAs increase patient involvement [50]. Using a DA as a source of information might differ from using social relationships for one. For future research, it is recommended examining whether a DA and informational support are complementary to each other, or substitute each other, in regard to the attitude towards taking an active role in medical decision-making.

The strengths of the study are the large sample size, the response rate of 70 % and the fact that the questionnaire was both sent through the internet and by post. However, the respondents were not fully representative of the Dutch population aged 18 years and older. Compared to this population, older people (≥ 65 years) are overrepresented. We expect that this does not affect our regression results, since all subgroups are of sufficient size to perform association analyses. Nevertheless, it can be argued that members of a health care panel are more interested in health care and therefore might have a more positive attitude towards taking an active role in medical decision-making. In the questionnaire, we provided a hypothetical situation to the respondents. We only informed them that it was not about common complaints, but about more severe ones. This could limit the degree to which our findings are generally applicable. The reason for this is that participation preferences are expected to be different depending on the nature of a particular decision [37]. For example, the type of care on which to decide upon, for example medical care and home care, has an impact upon the importance people attach to shared decision-making, as well as their actual involvement in decision-making [51]. Research also shows that diagnosis may affect patients’ preferences [10]. Some studies show that patients with a severe illness are less likely to prefer involvement than patients who are less ill, while others show the opposite [10]. Furthermore, we examined an attitude towards behaviour, instead of actual behaviour. Also the physicians’ role in the decision-making process was not included in this study. It remains unsure from this study what decisions people would make in real life, and whether they actually use their available support.

A limitation might also be that we excluded respondents who did not fill out all propositions concerning the emotional support available (*N* = 24; 1.8 %), the lay informational support available (*N* = 31; 2.4 %), or the medical informational support available (*N* = 154; 11.8 %). We performed sensitivity analyses in which we interpreted missing as the answer option ‘nobody’. The sensitivity analyses showed the same results. As mentioned, the available measurements of social support did not fit within the context of our study. We therefore believe that our measurement provides a starting point for the further development of measurements of social support in the context of medical decision-making. With regards to the availability of lay informational support, a limitation is that we had no insight into the person meant by the ‘someone else’ response. We did not include the concept of instrumental support as in the Netherlands costs for most medical care, medicines and medical devices are covered by the basic health insurance package [25], and also because there is a low level of out-of-pocket payments [26]. Nevertheless, lack of instrumental support (e.g. money or transportation) might be a barrier for people to take an active role in medical decision-making, particularly in other countries. We therefore recommend further research to also include the concept of instrumental support. A final limitation is that our data were obtained using a cross-sectional study design, and as such cannot provide any information about causal relationships.

## Conclusions

This study provides further insight into circumstances under which patients have a positive attitude towards taking an active role in medical decision-making. We found that people who have more medical informational support available have a more positive attitude towards taking an active role in medical decision-making. The availability of emotional support is only positively associated with the attitude towards taking an active role in medical decision-making among people who have a low level of education. This study shows that social resources are related to the attitude towards taking an active role in medical decision-making. Strategies aimed at increasing patient involvement have to address this.

## Abbreviations

DA: Decision aid
GP: General practitioner
GPs: General practices
